# Prognostic value of left ventricular peak strain dispersion for cardiovascular events in patients undergoing maintenance haemodialysis: a single-center retrospective cohort study with propensity score matching

**DOI:** 10.1080/0886022X.2026.2697546

**Published:** 2026-07-31

**Authors:** Wei Ding, Haohui Zhu, Shiqi Yu, Kaikai Shen, Yujiao Chai, Jianjun Yuan, Liyun Zhang, Yu Lu

**Affiliations:** a Department of Ultrasound, Henan Provincial People’s Hospital, Zhengzhou, Henan, China; b Henan University of Chinese Medicine, Zhengzhou, Henan, China; c China-Japan Union Hospital of Jilin University, Changchun, Jilin, China

**Keywords:** Haemodialysis, speckle-tracking echocardiography, peak strain dispersion, cardiovascular events, propensity score matching, cox regression

## Abstract

This study evaluates the association between baseline left ventricular (LV) peak strain dispersion (PSD) and 3-to-5-year cardiovascular outcomes in patients undergoing maintenance hemodialysis (MHD). This single-center retrospective cohort study screened 586 adult patients undergoing MHD at a tertiary renal unit between January 2018 and December 2020, with follow-up through June 2024. Patients were stratified by baseline PSD at the prespecified cutoff of ≥65 ms. One-to-one propensity score matching (nearest neighbor, caliper: 0.02) using 24 covariates was applied to minimize confounding. The primary outcome was major adverse cardiovascular events (MACE)—a composite of cardiovascular death, nonfatal myocardial infarction, hospitalization for heart failure, nonfatal stroke and sustained ventricular arrhythmia. Secondary outcomes included cardiovascular and all-cause mortality. Survival was analyzed using Kaplan–Meier curves, matched-pair robust Cox proportional hazards models and competing-risk sensitivity analyses. After matching, 336 patients (168 per group) were analyzed, with excellent covariate balance (all standardized mean differences <0.10). Over a median follow-up of 46.8 months, MACE occurred in 64 patients (38.1%) with high PSD and 34 patients (20.2%) with low PSD. High PSD was associated with a higher hazard of MACE (adjusted hazard ratio [aHR]: 2.05, 95% confidence interval [CI]: 1.48–2.84; *p* < 0.001), cardiovascular mortality (aHR: 2.18, 95% CI: 1.08–4.40; *p* = 0.030) and all-cause mortality (aHR: 1.58, 95% CI: 1.02–2.45; *p* = 0.041). Subgroup analyses demonstrated directionally consistent associations, with a stronger association in patients with impaired global longitudinal strain (interaction *p* = 0.03). Baseline LV PSD was associated with adverse cardiovascular outcomes in patients receiving MHD. Incorporating PSD into echocardiographic risk stratification may refine risk assessment, but multicenter prospective validation is required before threshold-based clinical implementation.

## 1. Introduction

Cardiovascular disease accounts for approximately 40–50% of deaths in patients undergoing maintenance hemodialysis (MHD), and the relative risk of cardiovascular mortality in this population is 10–20 times that of the age-matched general population [1,2]. Despite improvements in dialysis technology and pharmacological therapy, the burden of major adverse cardiovascular events (MACE) in MHD remains substantial. Conventional echocardiographic measures—most notably left ventricular ejection fraction (LVEF)—can underestimate risk because systolic dysfunction often develops late in the uremic cardiomyopathy trajectory [3,4].

Speckle-tracking echocardiography has emerged as a sensitive, angle-independent method to detect subclinical myocardial dysfunction. Global longitudinal strain (GLS) has demonstrated robust prognostic value in MHD, outperforming LVEF in the prediction of cardiovascular mortality [5,6]. However, GLS reflects averaged regional contraction and may mask heterogeneity in electromechanical activation. Peak strain dispersion (PSD), also referred to as left ventricular (LV) mechanical dispersion, is calculated as the standard deviation of the time from the electrocardiographic Q or R wave to peak negative longitudinal strain across 16–18 LV segments. Peak strain dispersion quantifies the temporal heterogeneity of regional myocardial deformation and has been associated with myocardial fibrosis burden and arrhythmic or ischemic outcomes in hypertrophic cardiomyopathy, post-infarct states, severe aortic stenosis, mitral valve prolapse and stable coronary artery disease [7–13]. Parallel advances in automated and artificial intelligence (AI)-assisted strain quantification have improved reproducibility and may facilitate larger external validation cohorts, though such tools still require disease-specific evaluation before routine clinical adoption [14,15].

Mechanistically, patients undergoing MHD are highly susceptible to dispersion-type injuries. Repetitive volume shifts, uremic toxins, mineral bone disorder, chronic inflammation and accelerated interstitial fibrosis combine to produce patchy myocardial scarring, LV hypertrophy and electromechanical dispersion before LVEF declines [16,17]. Hensen et al. reported an association between LV PSD and ventricular arrhythmia in patients undergoing dialysis and predialysis, suggesting that dispersion captures arrhythmogenic substrates not identified by ejection fraction [18]. A more recent longitudinal study of dialysis-dependent patients with preserved LVEF also suggested that higher peak strain dispersion is associated with worse long-term survival [19]. Nevertheless, available studies remain limited by modest sample sizes, single-center designs, short follow-up periods and the incomplete control of baseline confounding between patients with high versus low dispersion.

To address these limitations, our team undertook a single-center retrospective cohort study incorporating propensity score matching to balance baseline confounders and evaluated the association between baseline LV PSD and 3-to-5-year cardiovascular outcomes in patients undergoing MHD. We hypothesized that elevated PSD (≥65 ms) would be associated with a higher risk of MACE, cardiovascular mortality and all-cause mortality and would provide prognostic information beyond that supplied by LVEF and GLS.

## 2. Methods

### 2.1. Study design and setting

This single-center retrospective cohort study was conducted at a tertiary referral renal dialysis unit. Consecutive adult patients initiating or continuing MHD between 1 January 2018 and 31 December 2020 were screened, with follow-up through 30 June 2024. The study was approved by the institutional review board of the participating center, which waived the requirement for individual informed consent because only routinely collected, de-identified clinical data were used. An English summary translation of the ethics approval document is provided as Supplementary File 1 for editorial transparency. Reporting was conducted in accordance with the Strengthening the Reporting of Observational Studies in Epidemiology (STROBE) statement for observational studies and the Reporting of Studies Conducted Using Observational Routinely Collected Health Data for Pharmacoepidemiology (RECORD-PE) extension for studies using routinely collected health data [20,21]. The analysis plan was registered internally before outcome data were unlocked and was consistent with current guidance on the conduct and reporting of propensity score analyses in cardiovascular research [22].

### 2.2. Study population

The inclusion criteria were explicitly defined before data extraction as follows: patients (1) aged 18 years or older at baseline; (2) undergoing in-center hemodialysis treatment three times per week; (3) with a dialysis vintage of at least 3 months before index echocardiographic examination; (4) for whom complete digital apical four-, three- and two-chamber cine loops with electrocardiographic gating were available for speckle-tracking analysis; and (5) for whom baseline clinical, laboratory, dialysis and conventional echocardiographic covariates required for propensity score estimation were available. An index echocardiographic examination defined the study baseline. The exclusion criteria were as follows: (1) inadequate echocardiographic image quality (defined as fewer than 14 interpretable LV segments on apical views) precluding reliable speckle-tracking analysis; (2) patients with a history of hospitalization for MACE, including acute coronary syndrome, hospitalization for heart failure, stroke and sustained ventricular arrhythmia, or a documented LVEF below 40% within the preceding 6 months; (3) patients with an active malignancy who were receiving cytotoxic or radiation therapy; (4) patients with severe valvular heart disease, defined as aortic or mitral stenosis of at least moderate severity, severe valvular regurgitation or prosthetic valve implantation; (5) patients with congenital heart disease, hypertrophic or infiltrative cardiomyopathy or cardiac transplantation; and (6) patients who were pregnant or anticipating a renal transplantation within 12 months. These criteria were used to establish a cohort in which baseline PSD reflected stable MHD-related myocardial remodeling rather than acute cardiovascular instability or nonuraemic cardiomyopathy. Patients subsequently transitioning to peritoneal dialysis or receiving a successful transplant were censored at the date of modality change.

### 2.3. Echocardiographic assessment and peak strain dispersion

All baseline echocardiographic studies were performed according to a standardized unit protocol on the longest interdialytic day—typically 44–48 h after the preceding session—using commercially available ultrasound systems (Philips EPIQ 7 C or General Electric Vivid E95) according to joint American Society of Echocardiography and European Association of Cardiovascular Imaging recommendations [23]. This timing was selected because it was consistently available in the retrospective archive and allowed all patients to be assessed at the same point in the dialysis cycle. Postdialysis echocardiography was not routinely performed in this cohort and was therefore not included in the primary analysis. Two-dimensional speckle-tracking analysis of apical four-, three- and two-chamber views was performed offline using vendor-specific post-processing software, with frame rates maintained between 50 and 80 frames per second. Global longitudinal strain was calculated as the average peak longitudinal strain across 18 LV segments. Peak strain dispersion was defined as the standard deviation of the time interval between the onset of the electrocardiographic QRS complex and peak negative longitudinal strain across 16–18 interpretable segments, expressed in milliseconds, as is consistent with the method described by Haugaa et al. [8] and with contemporary scientific statement recommendations on speckle-tracking echocardiography [24]. A prespecified dichotomizing threshold of 65 ms was used based on prior cardiomyopathy literature, but this threshold was not treated as internally optimized for the population undergoing MHD. Echocardiographic parameters in patients receiving hemodialysis can differ before and after dialysis as loading conditions change [25], so the standardized archive-based timing should be considered when interpreting PSD values.

Only echocardiographic studies with archived digital cine loops, electrocardiographic timing and sufficient image quality for offline strain analysis were eligible. All included baseline examinations were reanalyzed by two experienced echocardiographers blinded to clinical outcomes, with discrepancies resolved by consensus before the database was locked. A random 10% sample was also re-read by an independent third reader to evaluate reproducibility. Intraclass correlation coefficients for intra- and inter-observer PSD measurements were 0.92 (95% CI: 0.87–0.95) and 0.88 (95% CI: 0.81–0.93), respectively.

### 2.4. Covariates and data extraction

Baseline covariates used for propensity score estimation were prespecified based on subject matter knowledge and published literature on cardiovascular risk in MHD. The 24 covariates comprised (1) demographic characteristics, including age, sex, body mass index, smoking history and the Charlson comorbidity index; (2) dialysis-related and haemodynamic parameters, including dialysis vintage, dialysis adequacy (expressed as Kt/V), interdialytic weight gain, predialysis systolic blood pressure and predialysis diastolic blood pressure; (3) comorbidities, including diabetes mellitus, hypertension and prior coronary artery disease; (4) laboratory values, including hemoglobin, serum albumin, corrected calcium, phosphate, intact parathyroid hormone, high-sensitivity C-reactive protein (hs-CRP) and N-terminal pro-B-type natriuretic peptide; and (5) conventional echocardiographic parameters, including LVEF, LV mass index, left atrial volume index, E/e’ ratio. Global longitudinal strain (GLS) was reserved as a separate strain-based covariate for outcome modeling and was therefore not counted among the 24 propensity-score covariates. Baseline PSD was the exposure variable and was also not counted as a covariate. Data were extracted from electronic health records and centralized in a structured research database with internal logic checks and source document verification on a 10% random audit sample. The analytic cohort was restricted to patients with complete baseline covariate data for these prespecified variables, and complete case analysis was therefore used for propensity score modeling. Multiple imputation was not performed.

### 2.5. Outcome ascertainment

The primary outcome was MACE, defined as the composite of cardiovascular death, nonfatal myocardial infarction (third universal definition criteria), hospitalization for decompensated heart failure, nonfatal ischemic or hemorrhagic stroke and sustained ventricular arrhythmia (ventricular tachycardia >30 s or ventricular fibrillation). Secondary outcomes were cardiovascular mortality (adjudicated by death certificates and hospital records) and all-cause mortality. Two board-certified cardiologists blinded to PSD values independently adjudicated all candidate events. Disagreements were resolved by consensus with a third reviewer, and inter-rater agreement (Cohen’s κ) was 0.89. Follow-up time was calculated from the baseline echocardiogram to the outcome event—death, transplantation, dialysis modality change, loss to follow-up or administrative censoring on 30 June 2024—that occurred first.

### 2.6. Propensity score matching

Because baseline PSD is not randomly assigned and is strongly correlated with age, comorbidity burden, LV geometry and inflammatory status, 1:1 propensity score matching was used to approximate the exchangeability between groups with high versus low PSD. Propensity scores were estimated using a multivariable logistic regression model with high PSD (≥65 ms) as the dependent variable and the 24 prespecified covariates listed in section 2.4 as independent variables. Matching was performed using nearest-neighbor matching without replacement and a caliper width of 0.02 on the logit of the propensity score, which is within the range recommended to minimize residual confounding while preserving sample size in cardiovascular observational studies [22,26]. Covariate balance was assessed using standardized mean differences, with values below 0.10 considered acceptable. To assess sensitivity to matching algorithm choice, 1:1 optimal matching and inverse probability of treatment weighting were also performed as prespecified sensitivity analyses.

### 2.7. Statistical analysis

Baseline characteristics are presented as mean ± standard deviation or median (interquartile range [IQR]) for continuous variables and as number (percentage) for categorical variables, with between-group comparisons made using a Student’s *t*-test, Mann–Whitney *U* test, chi-square (*χ*^2^) test or Fisher’s exact test, as appropriate. In the matched cohort, survival was visualized using Kaplan–Meier estimates, and between-group differences were tested using the log-rank test. Cox proportional hazards regression was used to estimate adjusted hazard ratios (aHRs) with 95% confidence intervals (CIs). To account for the matched design, all Cox models used robust sandwich variance estimation, clustered at the matched-pair level. The primary MACE model included a PSD group and a parsimonious set of clinically important covariates selected *a priori* to avoid overfitting. These included age, diabetes mellitus status, prior coronary artery disease, GLS, hs-CRP and serum albumin. Models for cardiovascular mortality and all-cause mortality used the same matched-pair robust variance structure but were interpreted cautiously because the number of death events was smaller. The proportional hazards assumption was verified using Schoenfeld residuals and log(−log) plots, and no meaningful violations were detected. Competing-risk sensitivity analyses were performed using Fine–Gray subdistribution hazard models, treating noncardiovascular death before MACE as a competing event. Prespecified subgroup analyses evaluated effect heterogeneity across age, sex, diabetes status, dialysis vintage, LVEF, GLS and hs-CRP strata, with likelihood ratio tests for interaction. Restricted cubic spline analyses with four knots at the 5th, 35th, 65th and 95th percentiles of PSD were used to explore the shape of the continuous PSD–outcome association.

A two-sided *P*-value <0.05 was considered statistically significant. Given the prespecified, hypothesis-driven nature of the subgroup tests, no formal adjustment for multiplicity was performed, and subgroup findings were interpreted as secondary. All analyses were conducted using R version 4.3.1 (R Foundation for Statistical Computing)—principally with the MatchIt, survival, survminer, cmprsk, sandwich and rms packages.

## 3. Results

### 3.1. Patient selection and covariate balance

**Figure 1.**
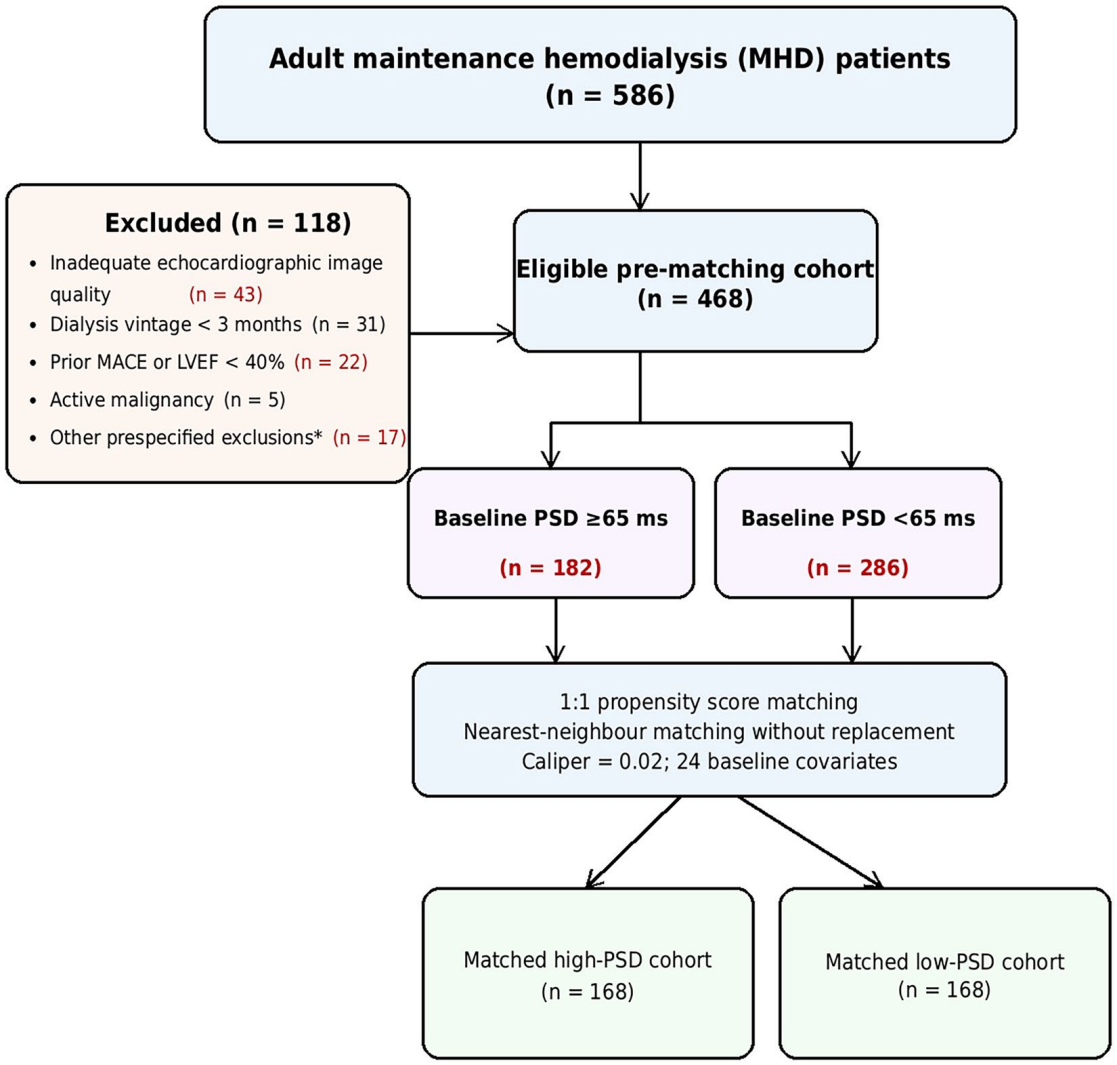
Study flow of patient selection, propensity score matching, and analytic plan.

Of the 586 patients receiving MHD screened, 468 met eligibility criteria and were included in the pre-matching analytic cohort (Figure 1). The reasons for exclusion were inadequate echocardiographic image quality (*n* = 43), a dialysis vintage of less than 3 months (*n* = 31), recent MACE or a recent LVEF below 40% (*n* = 22), active malignancies (*n* = 5) and other prespecified exclusions (*n* = 17). Baseline PSD was ≥65 ms in 182 patients (38.9%) and <65 ms in 286 patients (61.1%). Before matching, patients with high PSD were older, had a higher prevalence of diabetes mellitus and coronary artery disease, showed more pronounced LV remodeling and had higher hs-CRP concentrations than patients with low PSD (Table 1).

**Table 1.** Baseline characteristics of the unmatched and propensity score-matched cohorts.

Characteristic	Unmatched High PSD (*n* = 182)	Unmatched Low PSD (*n* = 286)	SMD (unmatched)	Matched High PSD (*n* = 168)	Matched Low PSD (*n* = 168)	SMD (matched)
Age, years, mean (SD)	62.8 (11.4)	57.2 (12.1)	0.48	61.9 (11.2)	61.4 (11.8)	0.04
Male sex, *n* (%)	108 (59.3)	165 (57.7)	0.04	99 (58.9)	98 (58.3)	0.02
Body mass index, kg/m², mean (SD)	23.8 (3.7)	23.5 (3.5)	0.08	23.7 (3.6)	23.6 (3.5)	0.03
Dialysis vintage, months, median (IQR)	48 (24–84)	36 (18–72)	0.22	46 (23–80)	44 (22–78)	0.03
Diabetes mellitus, *n* (%)	87 (47.8)	98 (34.3)	0.28	79 (47.0)	77 (45.8)	0.03
Hypertension, *n* (%)	165 (90.7)	241 (84.3)	0.20	151 (89.9)	153 (91.1)	0.04
Prior coronary artery disease, *n* (%)	48 (26.4)	42 (14.7)	0.29	41 (24.4)	39 (23.2)	0.02
Smoking history, *n* (%)	80 (44.0)	113 (39.5)	0.09	73 (43.5)	71 (42.3)	0.03
Charlson comorbidity index, median (IQR)	5 (4–7)	4 (3–6)	0.34	5 (4–7)	5 (3–6)	0.06
Predialysis systolic BP, mmHg, mean (SD)	148 (22)	143 (20)	0.24	146 (21)	145 (20)	0.05
Predialysis diastolic BP, mmHg, mean (SD)	82 (12)	78 (11)	0.35	80 (11)	80 (12)	0.04
Hemoglobin, g/L, mean (SD)	106 (14)	110 (13)	0.30	107 (14)	108 (13)	0.07
Serum albumin, g/L, mean (SD)	37.8 (4.1)	39.5 (3.8)	0.43	38.2 (4.0)	38.6 (3.9)	0.10
Phosphate, mmol/L, mean (SD)	1.82 (0.46)	1.76 (0.42)	0.14	1.80 (0.44)	1.79 (0.43)	0.02
Corrected calcium, mmol/L, mean (SD)	2.21 (0.22)	2.25 (0.21)	0.19	2.22 (0.22)	2.23 (0.21)	0.05
iPTH, pg/mL, median (IQR)	310 (188–520)	270 (150–460)	0.18	300 (180–500)	292 (170–492)	0.04
hs-CRP, mg/L, median (IQR)	4.8 (2.1–10.3)	2.6 (1.1–6.2)	0.46	4.3 (1.9–9.6)	4.1 (1.8–9.1)	0.04
NT-proBNP, pg/mL, median (IQR)	9,800 (4,800–18,500)	6,200 (2,800–12,800)	0.23	8,900 (4,400–17,200)	8,400 (4,000–16,800)	0.07
Kt/V, mean (SD)	1.42 (0.18)	1.45 (0.17)	0.17	1.43 (0.18)	1.44 (0.17)	0.06
Interdialytic weight gain, kg, mean (SD)	2.8 (0.9)	2.4 (0.8)	0.47	2.6 (0.8)	2.5 (0.8)	0.06
LVEF, %, mean (SD)	58.6 (7.2)	62.4 (6.1)	0.57	59.2 (7.0)	59.8 (6.7)	0.09
LV mass index, g/m², mean (SD)	128 (28)	112 (24)	0.61	125 (27)	123 (26)	0.08
Left atrial volume index, mL/m², mean (SD)	38 (10)	33 (9)	0.53	37 (9.6)	36 (9.3)	0.09
E/e′ ratio, mean (SD)	14.2 (4.1)	11.8 (3.6)	0.62	13.8 (3.9)	13.4 (3.7)	0.10
Global longitudinal strain, %, mean (SD)	−15.2 (3.4)	−18.4 (2.9)	1.01	−15.6 (3.3)	−15.9 (3.1)	0.09
Peak strain dispersion, ms, median (IQR)	74 (68–82)	52 (47–58)	—	73 (68–79)	53 (47–58)	—

SMD, standardized mean difference; PSD, peak strain dispersion; LVEF, left ventricular ejection fraction; hs-CRP, high-sensitivity C-reactive protein; iPTH, intact parathyroid hormone; NT-proBNP, N-terminal pro-B-type natriuretic peptide; Kt/V, dialysis adequacy.

**Figure 2.**
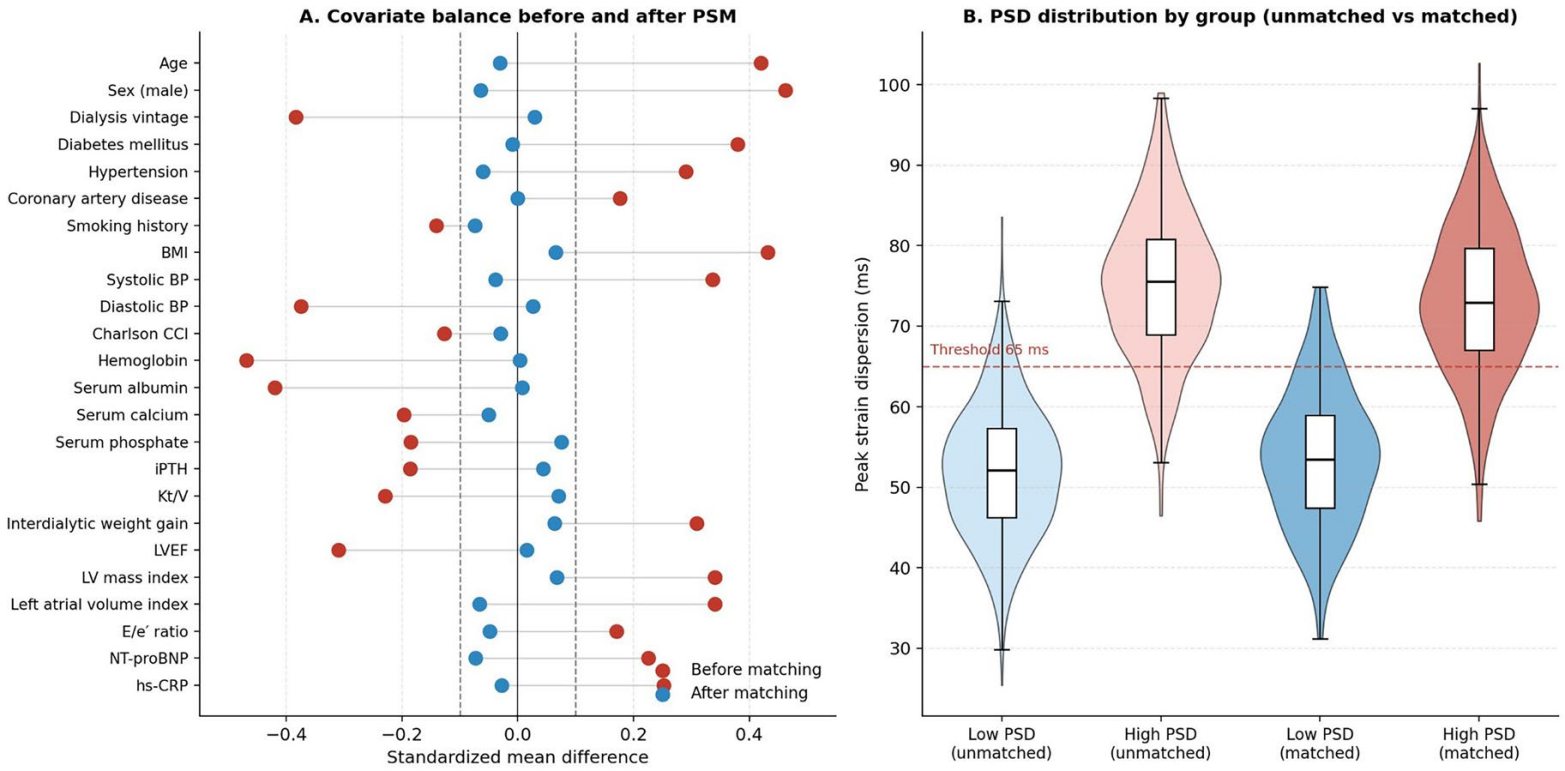
(A) Love plot of standardized mean differences for all 24 baseline covariates before and after propensity score matching; dashed lines denote the ±0.10 threshold for acceptable balance. (B) Distributions of baseline peak strain dispersion in the pre-matching and matched cohorts.

After 1:1 propensity score matching, 168 patients per group (336 total) were analyzed. Matching produced excellent covariate balance, with all 24 standardized mean differences reduced to below 0.10—a value in line with accepted thresholds for cardiovascular observational studies (Figure 2A) [22]. Table 1 presents the 24 prespecified matching covariates, GLS (reserved for outcome-model adjustment) and baseline PSD (the exposure variable), which explains the 26 descriptive rows in the table. The distribution of baseline PSD itself remained sharply separated between matched groups, confirming that matching reduced measured baseline confounding without collapsing the exposure contrast (Figure 2B). Median PSD in the matched high-PSD group was 72.8 ms (IQR: 68.4–79.1) versus 52.9 ms (IQR: 47.1–58.3) in the matched low-PSD group (*p* < 0.001).

### 3.2. Incidence of cardiovascular events

In the matched cohort, the median follow-up period was 46.8 months (IQR: 36.4–58.7). Major adverse cardiovascular events occurred in 98 patients (29.2%) − 64 (38.1%) in the high-PSD group versus 34 (20.2%) in the low-PSD group. Cardiovascular mortality occurred in 24 patients (14.3%) in the high-PSD group and 11 patients (6.5%) in the low-PSD group, and all-cause mortality occurred in 43 patients (25.6%) and 26 patients (15.5%) in the high- and low-PSD groups, respectively. Component-level events and crude incidence rates per 1,000 patient-years are provided in Table 2. Hospitalization for heart failure and sustained ventricular arrhythmia accounted for the largest absolute differences between groups.

**Table 2.** Component cardiovascular events and incidence rates in the matched cohort.

Event	High PSD (*n* = 168) *n* (%)	Low PSD (*n* = 168) *n* (%)	IR per 1,000 PY (High vs Low)	Unadjusted HR (95% CI)	Adjusted HR (95% CI)	*P* value
MACE (composite)	64 (38.1)	34 (20.2)	101.8 vs 47.2	2.14 (1.62–2.83)	2.05 (1.48–2.84)	<0.001
Cardiovascular death	24 (14.3)	11 (6.5)	31.1 vs 12.8	2.42 (1.49–3.94)	2.18 (1.08–4.40)	0.030
Non-fatal myocardial infarction	13 (7.7)	8 (4.8)	16.5 vs 9.8	1.85 (1.02–3.36)	1.67 (0.82–3.39)	0.157
Hospitalised heart failure	28 (16.7)	14 (8.3)	36.8 vs 16.9	2.18 (1.42–3.35)	1.98 (1.17–3.34)	0.011
Non-fatal stroke	12 (7.1)	8 (4.8)	15.7 vs 9.4	1.69 (0.93–3.07)	1.53 (0.73–3.19)	0.260
Sustained ventricular arrhythmia	9 (5.4)	3 (1.8)	11.5 vs 3.5	3.25 (1.38–7.66)	2.84 (1.02–7.90)	0.046
All-cause mortality	43 (25.6)	26 (15.5)	56.9 vs 31.4	1.82 (1.31–2.52)	1.58 (1.02–2.45)	0.041

IR, incidence rate; PY, patient-years; HR, hazard ratio; CI, confidence interval. Adjusted estimates were obtained from Cox models with robust sandwich variance clustered by matched pair. The primary MACE model included PSD group, age, diabetes mellitus, prior coronary artery disease, global longitudinal strain, hs-CRP, and serum albumin; mortality models used the same matched-pair robust variance structure and were interpreted cautiously because of fewer death events.

### 3.3. Kaplan–Meier survival analyses

**Figure 3.**
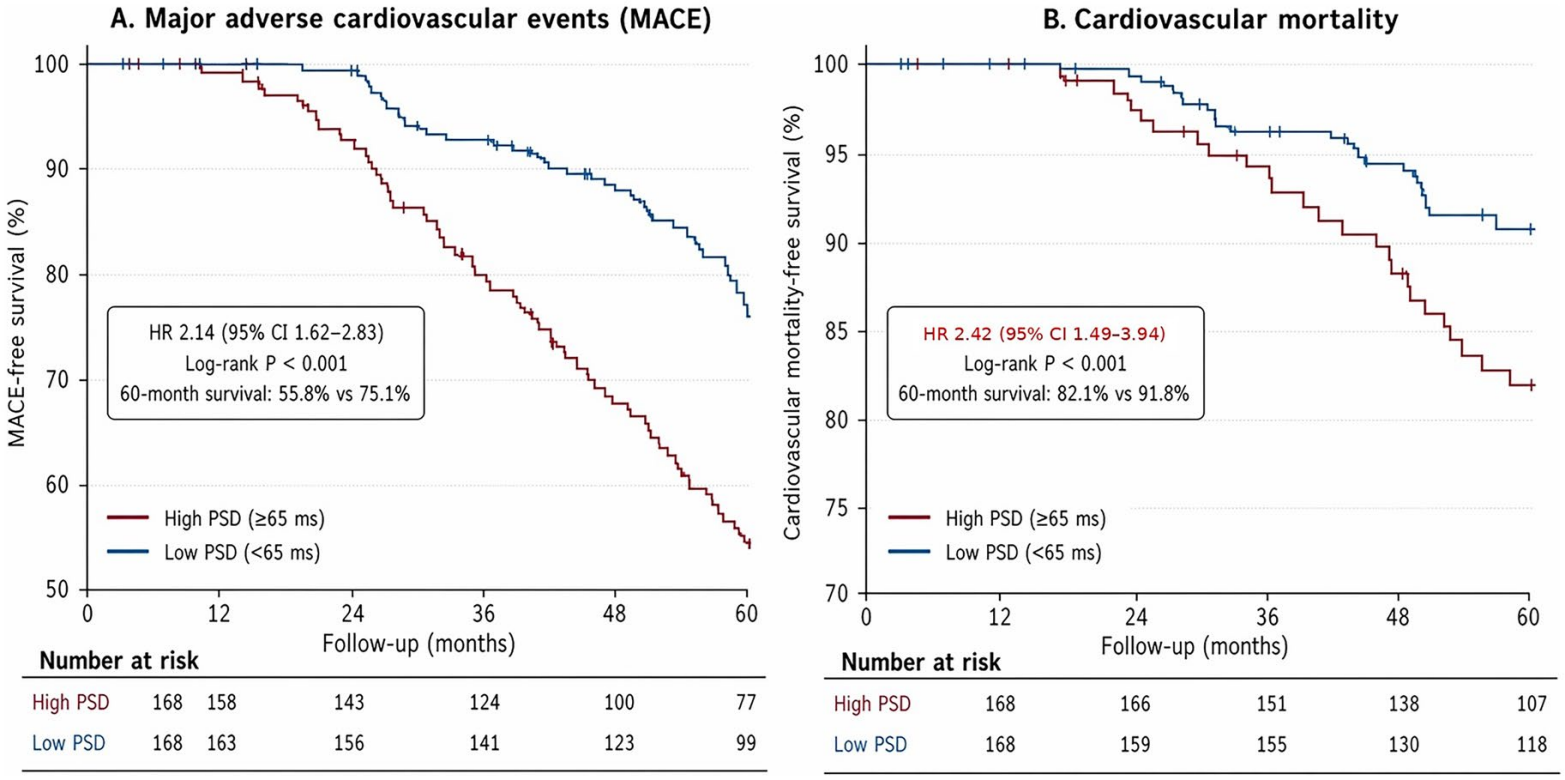
Kaplan–Meier curves for (A) major adverse cardiovascular events and (B) cardiovascular mortality in the propensity score-matched cohort, with number-at-risk tables below each panel. The number-at-risk values represent patients still under observation at each time point and are not cumulative event counts.

Kaplan–Meier estimates showed a clear separation between the survival curves of patients with high versus low PSD, which became apparent within the first 12 months and widened progressively during follow-up (Figure 3). The log-rank test was significant for MACE (*p* < 0.001; unadjusted hazard ratio [uHR]: 2.14, 95% CI: 1.62–2.83) and for cardiovascular mortality (*p* < 0.001; uHR: 2.42, 95% CI: 1.49–3.94). Sixty-month cumulative MACE-free survival was 55.8% (95% CI: 49.1–61.9) in the high-PSD group and 75.1% (95% CI: 68.7–80.3) in the low-PSD group. The corresponding 60-month cardiovascular mortality-free survival rate was 82.1% in the high-PSD group and 91.8% in the low-PSD group. The number-at-risk rows in Figure 3 denote patients remaining under observation at each landmark and therefore cannot be calculated as 168 minus the cumulative event count because censoring also reduces the risk set.

### 3.4. Independent association of peak strain dispersion with cardiovascular outcomes

**Figure 4.**
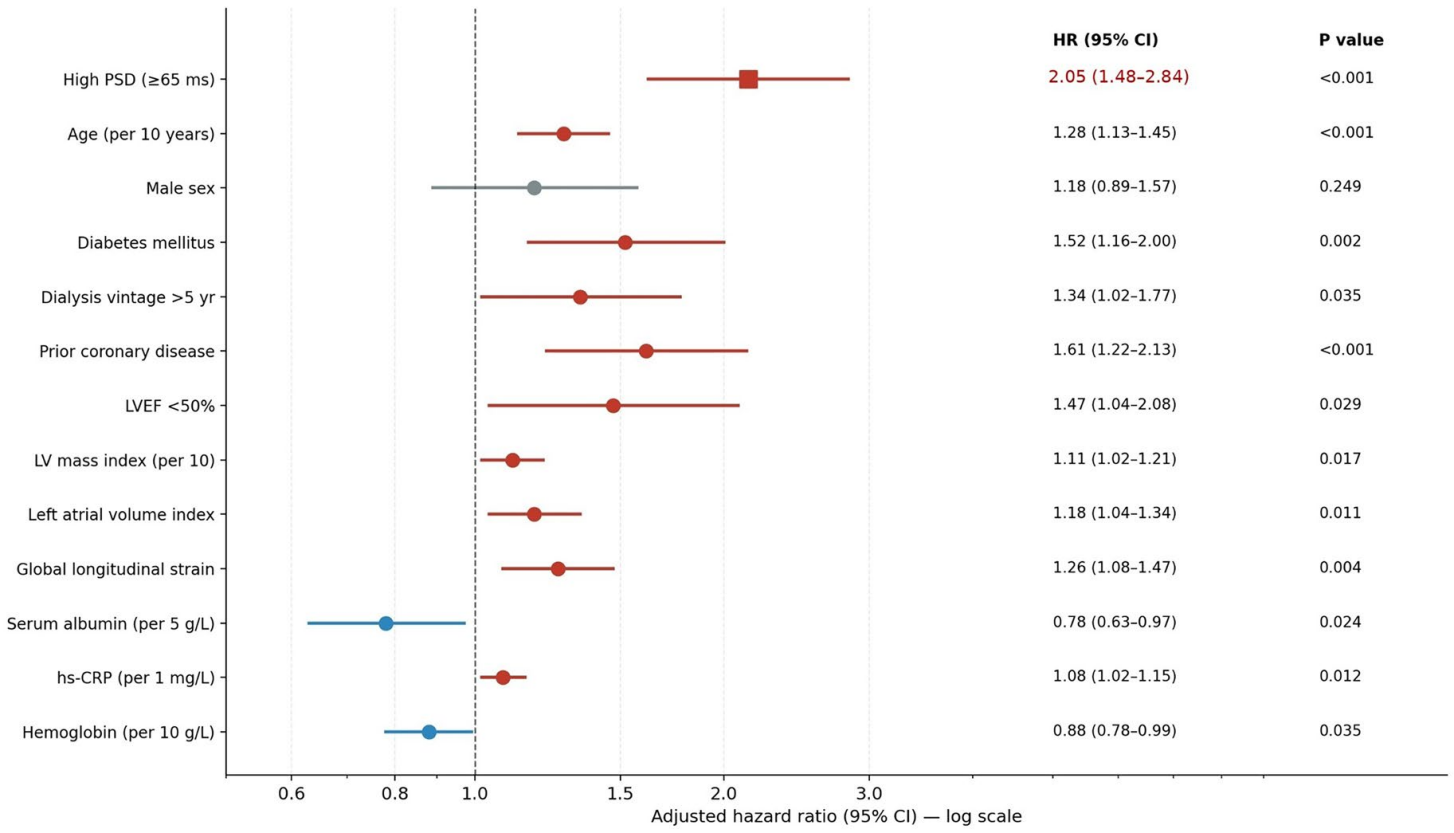
Forest plot of the multivariable matched-pair robust Cox proportional hazards model for major adverse cardiovascular events. Hazard ratios are displayed on a logarithmic scale. The red square denotes the primary exposure (high PSD).

After adjustment for the prespecified clinical covariates and the use of matched-pair robust variance estimation, high PSD remained associated with MACE (aHR: 2.05, 95% CI: 1.48–2.84; *p* < 0.001), cardiovascular mortality (aHR: 2.18, 95% CI: 1.08–4.40; *p* = 0.030) and all-cause mortality (aHR: 1.58, 95% CI: 1.02–2.45; *p* = 0.041). The full multivariable model for MACE is presented in Figure 4. Other factors associated with MACE included advancing age, diabetes mellitus, prior coronary artery disease, impaired GLS, elevated hs-CRP and lower serum albumin. An LVEF below 50% was only modestly associated with MACE after adjusting for PSD and GLS (aHR: 1.47, 95% CI: 1.04–2.08), which is consistent with prior data showing that strain-based parameters capture risks that ejection fraction does not [5,6]. Restricted cubic spline analysis supported a monotonic increase in MACE hazard with PSD continuously above approximately 55 ms; however, the 65-ms threshold should be interpreted as a prespecified risk stratification cutoff rather than an internally optimized decision threshold.

### 3.5. Subgroup and sensitivity analyses

**Figure 5.**
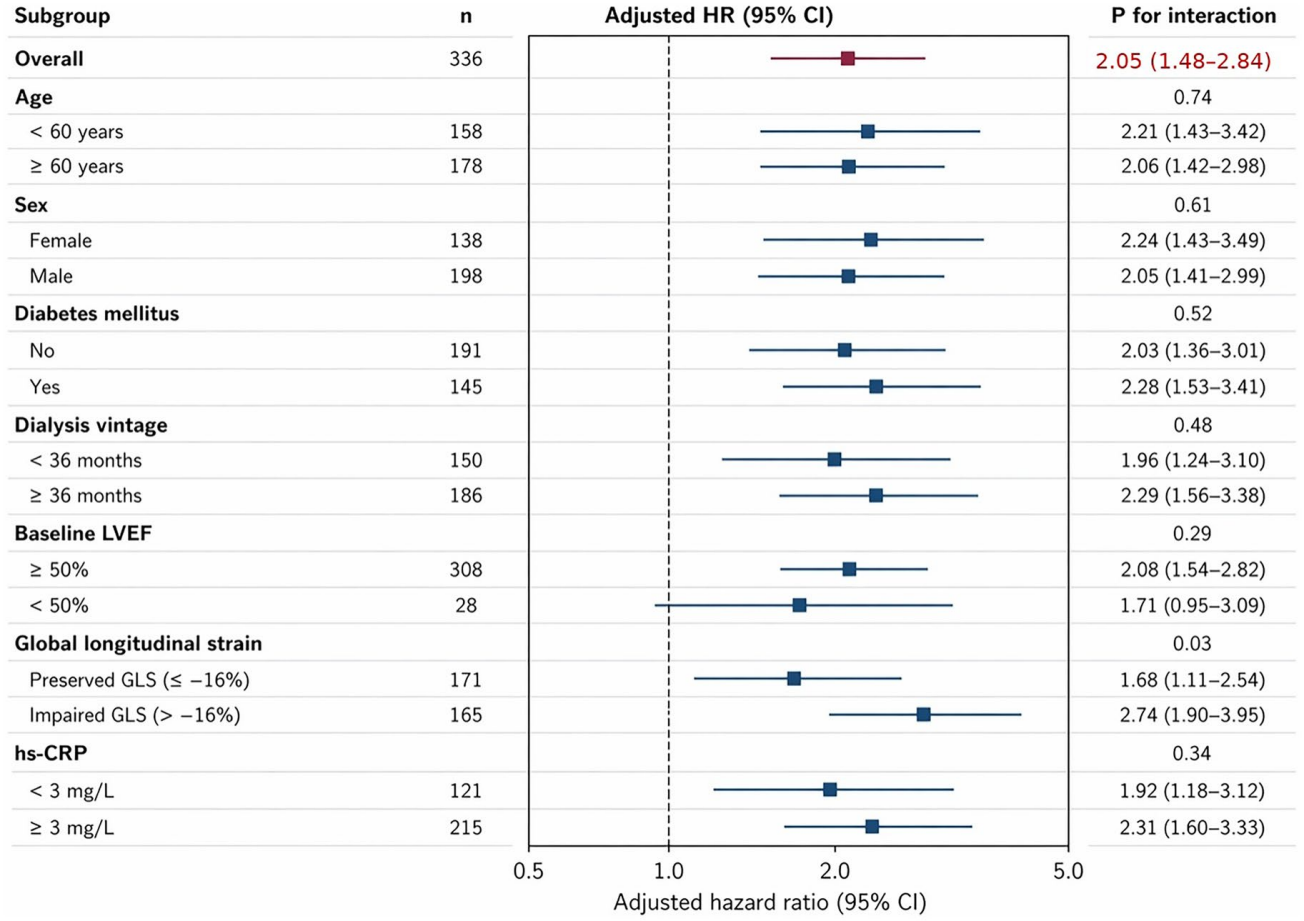
Forest plot of subgroup analyses for the association of high versus low PSD with major adverse cardiovascular events, with P-values for interaction across subgroups.

Prespecified subgroup analyses are shown in Figure 5. The association between high PSD and MACE was directionally consistent across all subgroups defined by age, sex, diabetes status, dialysis vintage, baseline LVEF, GLS and hs-CRP. Formal tests for interaction were non-significant for most strata, with a single statistically significant interaction observed for GLS (interaction *p* = 0.03), indicating a stronger association between high PSD and MACE among patients with impaired GLS (aHR: 2.74, 95% CI: 1.90–3.95) than among those with preserved GLS (aHR: 1.68, 95% CI: 1.11–2.54). Results were materially unchanged in sensitivity analyses using optimal matching (aHR for MACE: 1.98, 95% CI: 1.43–2.75) and inverse probability of treatment weighting (aHR: 1.94, 95% CI: 1.45–2.61) [27], as well as when patients with severe LV hypertrophy were excluded (aHR: 1.96, 95% CI: 1.39–2.76). In the Fine–Gray competing-risk model, high PSD remained associated with MACE when noncardiovascular death before MACE was treated as a competing event (subdistribution HR: 1.93, 95% CI: 1.38–2.71; *p* < 0.001).

## 4. Discussion

In this single-center retrospective cohort of nearly 500 patients undergoing MHD with long-term follow-up and rigorous propensity score matching, elevated baseline LV PSD was associated with a greater-than-twofold increase in the hazard of MACE and with higher hazards of cardiovascular and all-cause mortality. The association was consistent across prespecified subgroups, robust to multiple sensitivity analyses and largely independent of LVEF, GLS and conventional clinical risk factors. To the best of our knowledge, this is one of the few propensity-matched cohort studies specifically evaluating PSD as a prognostic marker in MHD, and it addresses important methodological limitations of earlier work in this population that was constrained by small sample sizes, shorter follow-up periods and incomplete control of baseline confounding [18,19].

Our findings extend the emerging evidence that markers of regional myocardial heterogeneity provide prognostic information that averaged strain parameters may miss. In hypertrophic cardiomyopathy, PSD has been associated with appropriate implantable cardioverter defibrillator therapy and sudden cardiac death [7,9]. Similar prognostic associations have been reported in ischemic cardiomyopathy, mitral valve prolapse, severe aortic stenosis and stable coronary artery disease [8,10–13]. The MHD phenotype shares several of the pathophysiological features that render PSD prognostically relevant in these conditions, including accelerated interstitial fibrosis, calcium–phosphate dysregulation, chronic inflammation, repetitive volume oscillation and autonomic imbalance. Each of these creates a substrate of patchy electromechanical delay that is well quantified by PSD but less visible in LVEF. The observation in our cohort that PSD retained prognostic value after adjustment for GLS suggests that dispersion captures complementary mechanistic information—namely, arrhythmogenic substrate and regional heterogeneity—rather than average contraction, which is consistent with contemporary scientific statement recommendations positioning PSD as an emerging supplementary strain marker [24].

The stronger association between PSD and MACE in patients with impaired GLS (interaction *p* = 0.03) is biologically plausible. Patients with lower GLS are further along the uremic cardiomyopathy trajectory, with greater cumulative fibrosis and more advanced remodeling. Superimposed heterogeneity in regional activation in this context may reflect additional arrhythmogenic or ischemic substrate. This aligns with prior evidence showing that PSD adds incremental value to global strain in risk prediction for ventricular arrhythmias and reverse remodeling [9,29]. Clinically, this suggests that PSD may be most useful as a risk-refining tool in patients with borderline or impaired GLS—a group in whom conventional echocardiographic metrics are often ambiguous for decision-making purposes around cardioprotective intensification.

From a clinical perspective, our findings support a cautious and practical risk-stratification pathway rather than an immediate treatment mandate. During routine echocardiographic surveillance in stable patients receiving MHD, PSD could be measured alongside LVEF, GLS, LV mass index and diastolic indices. Patients with a PSD below 65 ms would continue standard nephrology and cardiovascular surveillance, whereas those with a PSD above 65 ms would be considered for structured cardionephrological review; careful reassessment of volume and blood pressure control; optimization of renin–angiotensin system inhibitors; beta-blockers and mineralocorticoid receptor antagonists, where tolerated; review of dialysis prescription and dry weight; and targeted rhythm surveillance when symptoms, impaired GLS or structural heart disease coexist. Peak strain dispersion should not be used as a stand-alone indication for implantable cardioverter defibrillator therapy, but the difference in sustained ventricular arrhythmia is clinically relevant, given the persistent uncertainty about the benefit of a defibrillator in MHD [30].

Several aspects of our design strengthen confidence in these findings. Although single-centred, the moderate sample size (*n* = 468 included; 336 matched) remains larger than several earlier studies of PSD in dialysis-receiving populations [18,19]. The 1:1 propensity score matching using 24 covariates achieved stringent balance across measured confounders, outcomes were adjudicated by blinded cardiologists with high inter-rater agreement, follow-up was long (median: 46.8 months, extending to 60 months for some patients) and the findings were consistent across matching algorithms, inverse probability weighting and competing-risk sensitivity analyses [22,26,27]. Additionally, the analysis plan was specified before outcome data were unlocked, and reporting adhered to STROBE and RECORD-PE standards [20,21].

Several limitations should nonetheless be acknowledged. As a retrospective observational study, residual confounding by unmeasured factors, such as longitudinal medication adherence, inflammatory burden between baseline visits and detailed volume–status metrics, cannot be fully excluded even after propensity score matching. Propensity score methods balance only measured covariates, and the present design cannot approximate randomized trial-level evidence [27,28]. The cohort excluded patients with an LVEF below 40%, recent MACE or inadequate echocardiographic windows, which reduced acute confounding but also enriched the study sample for relatively stable patients receiving MHD with interpretable images. Thus, the findings may not apply to the full clinical spectrum of MHD (e.g. patients with advanced systolic dysfunction, recent decompensation or poor acoustic windows). As a single-center study, external generalizability to other dialysis units with different patient case mixes, dialysis protocols and imaging platforms remains to be demonstrated. Speckle-tracking strain is vendor-dependent, and the present study used only Philips and General Electric platforms with vendor-specific post-processing. Cross-vendor reproducibility testing was not performed, and absolute PSD values may not be directly transferable to centers using other equipment. The chosen cutoff of 65 ms was prespecified from prior cardiomyopathy literature and supported by exploratory spline patterns, but it was not externally validated in an independent MHD cohort and should not be interpreted as a universal treatment threshold. Postdialysis echocardiography was not routinely available, so the stability of PSD immediately after dialysis could not be formally assessed. Finally, although Fine–Gray models addressed competing noncardiovascular death as a sensitivity analysis, the relatively small number of cardiovascular deaths limited precision for mortality-specific estimates.

Future work should focus on the prospective, multicenter validation of PSD-based risk stratification across dialysis settings, ultrasound vendors and post-processing platforms. Larger cohorts should evaluate whether PSD improves discrimination, calibration and clinical utility when added to composite risk scores that include natriuretic peptides, cardiac troponin, LV mass, GLS and dialysis-related volume metrics. Integration with wearable arrhythmia monitoring and AI-assisted strain workflows may further refine the identification of patients receiving MHD who are at imminent risk, but such approaches should follow contemporary reporting and validation standards for prediction models [14,15,31]. Cardiac magnetic resonance imaging with late gadolinium enhancement and T1 mapping could clarify the degree to which PSD reflects interstitial fibrosis in uremic cardiomyopathy, paralleling findings in hypertrophic cardiomyopathy [32].

## 5. Conclusion

In this single-center propensity-matched cohort of patients receiving MHD, elevated baseline LV PSD was associated with MACE, cardiovascular mortality and all-cause mortality over 3–5 years of follow-up. The association was robust after extensive measured-confounder adjustment, matched-pair variance estimation and competing-risk sensitivity analysis, and it was directionally consistent across clinically important subgroups. Peak strain dispersion offers prognostic information beyond LVEF and GLS and, pending external multicenter and prospective validation, may serve as a practical echocardiographic marker to refine cardiovascular risk assessment in stable patients undergoing MHD.

## Supplementary Material

Supplemental Material

## Data Availability

All data generated or analyzed during this study are included in the article.
