# Supplementary material for: Prognostic value of left ventricular peak strain dispersion for cardiovascular events in patients undergoing maintenance haemodialysis: a single-center retrospective cohort study with propensity score matching
Source: Ren Fail. 2026 Jul 31;48(1):2697546. doi: 10.1080/0886022X.2026.2697546 (PMC13431026; doi:10.1080/0886022X.2026.2697546)
Supplement: Supplemental Material [file IRNF_A_2697546_SM7623.docx]

Supplementary File 1. English summary translation of ethics approval

The institutional review board of the participating tertiary renal dialysis centre reviewed and approved this single-centre retrospective cohort study. The study used routinely collected, de-identified clinical, dialysis, laboratory, and echocardiographic data from adult maintenance hemodialysis patients screened between 1 January 2018 and 31 December 2020, with follow-up through 30 June 2024. Because the study was retrospective, involved no additional patient contact or intervention, and used anonymised data for analysis, the requirement for individual informed consent was waived by the institutional review board. The study was conducted in accordance with the principles of the Declaration of Helsinki and applicable institutional regulations for retrospective clinical research.
